# Large-scale production of recombinant human lactoferrin from high-expression, marker-free transgenic cloned cows

**DOI:** 10.1038/s41598-017-11462-z

**Published:** 2017-09-06

**Authors:** Ming Wang, Zhaolin Sun, Tian Yu, Fangrong Ding, Ling Li, Xi Wang, Mingbo Fu, Haiping Wang, Jinming Huang, Ning Li, Yunping Dai

**Affiliations:** 1 State Key Laboratory for Agrobiotechnology, College of Biological Sciences, China Agricultural University, Beijing, China; 2 Kejienuo Biotechnology Company, Wuxi, China; 3 Dairy cattle Research Center, Academy of Agricultural Sciences, Shandong, China

## Abstract

Human lactoferrin (hLF) is a valuable protein for pharmaceutical products and functional foods, and worldwide demand for this protein has steadily increased. However, large-scale recombinant human lactoferrin (rhLF) production using current animal bioreactor techniques is limited by the low expression of foreign proteins, the use of antibiotic resistance genes and the down-regulation of endogenous milk proteins. Here, we generated a herd of marker-free, hLF bacterial artificial chromosome (BAC) transgenic cloned cows, as confirmed by Polymerase chain reaction, Southern blot and Western blot analyses. These transgenic cloned cows produced rhLF in milk at concentrations of 4.5–13.6 g/L. Moreover, the total protein content of the milk was increased. Over two hundred transgenic cloned cows were propagated by multiple ovulation and embryo transfer (MOET). A total of 400–450 g of rhLF protein, which shows similar enzymatic activity to natural hLF in iron binding and release, can be purified on a large scale from >100 L of milk per day. Our results suggested that transgenic bovine mammary bioreactors have the potential for large-scale protein production.

## Introduction

Human lactoferrin (hLF) is a 79 kDa multifunctional glycoprotein involved in intestinal iron absorption and the non-specific immune system^1–3^. In view of its medicinal value and the global demand for hLF, the large-scale production of functional recombinant hLF (rhLF) has become a major goal. The bovine mammary bioreactor may be an excellent system for the large-scale production of rhLF due to its large capacity for protein synthesis, efficient production, and low cost relative to those of *in vitro* fermentation and tissue culture systems^4^.

The expression level of rhLF in previous bovine mammary bioreactors has been low (0.2–2 g/L). The low efficiency of this transgenic bioreactor is attributable to the pronuclear microinjection technique used and the long waiting period required to establish transgenic animal lines^5^. Although our group has recently improved this technology and has generated hLF bacterial artificial chromosome (BAC) transgenic cloned cows by somatic cell nuclear transfer (SCNT)^6^, the expression level of rhLF in these cows was 3 g/L, which is not much higher than those of previous studies^5^. Thus, improving the expression levels of recombinant protein in bovine mammary bioreactors is crucial. Antibiotic-selectable marker genes have been widely used to generate transgenic animal mammary bioreactors^7–9^. However, the presence of foreign marker genes interferes with the expression of neighbouring endogenous genes and hampers phenotypic and genetic analyses^10, 11^. This approach may also create public concerns regarding biological safety. Thus, marker-free technology must be used to generate transgenic animals.

Importantly, previous reports have shown that recombinant proteins compete for production in the mammary glands of transgenic animals^12, 13^. Although exogenous β-lactoglobulin (BLG) and rhLF proteins are highly expressed at 30 g/L in transgenic mice and goats, the endogenous milk proteins are down-regulated, and the total protein level is not increased. The ceiling effect for protein production in transgenic animal mammary glands remains to be fully investigated, especially in cattle^5, 6, 13^. Finally, the large-scale production of a biological protein in the animal mammary gland using a herd of a few hundred transgenic cloned cattle has thus far been poorly investigated. These disadvantages have limited the applications of transgenic animal bioreactors.

Here, we established a simple and safe method based on nucleofection^14^ and single-cell limited dilution^15, 16^ to generate marker-free hLF BAC transgenic cows that produced a high level (4.5–13.6 g/L) of functional rhLF. To the best of our knowledge, this study reports the highest levels of expression of rhLF in marker-free transgenic cows. The total protein of the milk was significantly increased. Additionally, a herd of two hundred transgenic cattle was established by multiple ovulation and embryo transfer (MOET). A total of 400–450 g of recombinant protein can be purified from >100 L of milk per day.

## Methods

### Ethics statement

All transgenic cows were fed the same standard diet and raised under the same conditions. All procedures were guaranteed by an animal welfare agency and were in accordance with the approved guidelines of the China Council on Animal Care and Protocols. All protocols involving the use of animals were in accordance with the approved guidelines of the Institutional Animal Care and Use Committee of the China Agricultural University (Permit Number: SKLAB-2012-06-01).

### Preparation of the marker-free hLF BAC vector

A marker-free hLF BAC vector containing the entire hLF genomic sequence without an antibiotic resistance marker was obtained in our previous study, which contains the procedural details^17^. The marker-free hLF BAC vector was purified using the QIAGEN Large-Construct Kit (catalogue no. 12462; Qiagen, Germany). After enzyme digestion by NotI and pulsed-field gel electrophoresis with a CHEF Mapper III (Bio-Rad, Hercules, CA, USA), a linearized 150 kb vector containing the entire hLF genomic sequence was separated from the marker-free hLF BAC vector.

### Nucleofection of the marker-free hLF BAC vector into bovine foetal fibroblasts and identification of single-cell colonies

Primary bovine foetal fibroblast (BFF) cells were isolated from Holstein cattle foetuses by disaggregation of the entire body without the head or viscera and were cultured in Dulbecco’s modified Eagle’s medium (DMEM) (Gibco, Grand Island, New York, USA) supplemented with 10% foetal bovine serum (FBS) (Gibco) at 37.5 °C in 5% CO_2_ and humidified air. The 2 µg linearized marker-free 150 kb hLF fragment was nucleofected into 1 × 10^6^ BFFs using Amaxa Nucleofector reagent (Lonza Group AG, Basel, Switzerland) according to the manufacturer’s guidelines, and the T-016 programme was selected. Limited dilution was used in the cell colonies that formed 24–48 h after transfection, and the cell concentration was approximately 500 cells/dish (10 cm^2^). Individual cell clones were isolated 5–7 days after culture dilution, and the clones were then expanded in culture, sequence analysed and cryopreserved after a total of 12–14 days in culture. PCR was performed to further screen for positive cells using the P1 (5′-GTGGGTATTTGACAAGGGGT-3′) and P2 (5′-CAACTCTAACCATCGGACCT-3′) primers with a programme of 35 cycles of 94 °C for 30 s, 60 °C for 30 s, and 72 °C for 30 s, followed by holding at 72 °C for 10 min. For primers P3 (5′-GGCTCTGAGCAAATGCTACC-3′) and P4 (5′-GGAAGAACCTGACCATTCCA-3′), P5 (5′-TTGACTGGATCCCGTAGAGG-3′) and P6 (5′-ATGGGTGGGTTCTGAGACTG-3′), P7 (5′-CTCAAAGAAAGTCCCAACCC-3′) and P8 (5′-GTCTCTTGGATGCGTTGCC-3′), P9 (5′-GATGCTGTGACCCTTGATGG-3′) and P10 (5′-TGCAGGAGAGACTCTGTCATG-3′), PCR was performed for 35 cycles of 94 °C for 30 s, 58 °C for 30 s, and 72 °C for 30 s, followed by holding at 72 °C for 10 min.

### SCNT

The SCNT procedure was performed as previously described^18^. Briefly, the nuclei of transgenic cells were transferred into enucleated oocytes to produce reconstructed embryos *in vitro* using the ECM® 2001 Electro cell manipulation system (BTX, San Diego, CA, USA). Day 7 blastocysts were collected for future transplantation. A total of 492 blastocysts were transferred into 328 recipient Chinese Luxi yellow cows. For each recipient, we transferred 1–2 transgenic cloned blastocysts. Pregnancy was detected by ultrasonography at 60 days and 180 days post-transfer. All experiments were performed in accordance with the relevant guidelines and regulations, and the Institutional Animal Care and Use Committee of China Agricultural University approved this research.

### Southern blot analysis

Genomic DNA was obtained from the ear tissue of transgenic and wild-type (WT) cows, extracted using phenol/chloroform, and then digested (10 µg) with the restriction enzyme EcoRI overnight. A digoxigenin-labelled probe targeting the hLF gene was amplified using a PCR DIG Probe Synthesis Kit (Roche Mannheim, Germany) and the primer pair hLF-F: 5′-CCCGTCATGTACTAAATCCTT-3′ and hLF-R: 5′-GTAAGTCAGTGTCA

### **ATGGGG**A-3′

After 0.7% agarose gel electrophoresis for 4 h, the DNA was transferred to a nitrocellulose membrane filter (Roche, Mannheim, Germany) for blotting. The nitrocellulose membrane filter was hybridized with a probe for 18 h and then incubated with a primary antibody for 0.5 h. The positive bands were expected to be approximately 2.2 kb in size.

### Quantitative PCR (qPCR)

A qPCR assay was used to detect the transgene copy number. The primer pair P-QhLF-F/R (5′-GATGAAGGGGGAGTATGGCAG-3′ and 5′-CATCCCC TTATGGCGAGAGCC-3′), which amplifies a 110 bp product, was used to calculate the transgene copy number in the transgenic cows. The primer pair bovine myostatin F/R (bovine myostatin F: 5′-TCCGTCCTGGCGTGGTAG-3′; bovine myostatin R: 5′-GCTATCAGA CAACTTTTGCCCAAG-3′), which amplifies a 122 bp product from the myostatin gene (GenBank: JQ711180.1), was used as an internal control. Each PCR amplification was performed in a 20 μL reaction volume containing 1 μL of template DNA (10 ng/μL), 0.3 μL of each primer, 10 μL of Power SYBR Green Mix, and 8.4 μL of distilled-deionized water and run under the following conditions: initial denaturation at 95 °C for 10 min; 40 cycles of denaturation (95 °C for 10 s), annealing (60 °C for 10 s), and extension (72 °C for 10 s); and a melting curve generated by treatment at 95 °C for 5 s, 65 °C for 1 min, and then 97 °C continuously. A gradient dilution of the vector DNA mixed with WT DNA (10 ng) was used to produce a standard curve, with SYBR Green functioning as the fluorescent dye. All PCR reactions were performed using the Roche LightCycler 480 system (LC 480; Roche Diagnostics, Basel, Switzerland).

### Collection and total protein detection of transgenic milk

To induce lactation, we intramuscularly injected medroxyprogesterone acetate (25 mg/kg/day) and oestradiol benzoate (7.5 mg/kg/day) into eight-month old transgenic cloned cows for seven days. Milk samples were collected for at least two weeks from the first day of lactation. The total protein in the milk of each cow at each collection time was detected by the MilkoScan^TM^ Minor analyser (FOSS, Hillerod, Denmark).

### Analysis of rhLF from the milk of transgenic cows

For SDS-PAGE, milk protein samples were separated on 8% Tris-glycine polyacrylamide gels under denaturing and reducing conditions, and the protein content was quantified by staining with Coomassie brilliant blue dye. For western blot analysis, the diluted milk samples were separated on 8% Tris-glycine polyacrylamide gels under denaturing and reducing conditions and were then transferred to polyvinyl difluoride membranes (Invitrogen Corporation, Carlsbad, CA, USA), which were then incubated with a polyclonal anti-human hLF antibody (dilution, 1:2,000; United States Biological, Inc., Swampscott, MA, USA) followed by a horseradish peroxidase-conjugated secondary anti-rabbit IgG antibody (dilution, 1:20,000; Sino-American Co., Beijing, China). The rhLF expression levels were measured using a Human Lactoferrin Enzyme-linked Immunosorbent Assay (ELISA) Kit (Bethyl Laboratories, Inc., Montgomery, TX, USA).

### Purification of rhLF

The milk was centrifuged at 2500 rpm for 20 min at 4 °C to remove the fat. The skimmed milk was acidified to pH 4.6 to precipitate casein and was centrifuged at 100,000 × *g* at 20 °C for 1 h. The purification procedure was performed using an ÄKTA pure system (GE Healthcare, Uppsala, Sweden). First, after equilibrating three columns with equilibration buffer (20 mM phosphate buffer (PB), pH 7), the samples (100 L) were loaded onto a BPG 300/500 column, and the protein was eluted with 0.4 M NaCl and 1 M NaCl. Next, an Ultracel-30 membrane (Millipore Corporation, Billerica, MA, USA) was used to concentrate the fractions containing rhLF on an ÄKTA Crossflow automated cross-flow filtration system (GE Healthcare, Uppsala, Sweden). After purification, the rhLF was exchanged with 20 mM PB, and the quantity and quality of the purified rhLF were detected by SDS-PAGE.

### Iron binding and iron desaturation analysis on rhLF

To measure the iron-binding properties of rhLF at different Fe concentrations, the purified protein was saturated with freshly prepared FeCl_3_·6H_2_O solution based on a previous protocol^6^. Briefly, 5 ml of rhLF (20 mg/mL) was dissolved in 0.1 M NaCl, then mixed with 1 ml of FeCl_3_·6H_2_O (1–10 mM) and 4 ml of NaHCO_3_ (250 mM) and incubated at room temperature for 1 h. Subsequently, the blank control and sample solution were passed through a 0.2 µm filter. The absorbance at 465 nm was measured on a Lincam spectrophotometer (Lincam, Cambridge, UK). The concentration (%) of iron-saturated rhLF was calculated as (OD (sample)-OD (blank)/0.57) × 100. Commercially available hLF and bovine lactoferrin (bLF) were also measured as controls. To measure the iron-releasing properties of rhLF under different pH conditions, iron-saturated LF was made by combining 5 ml of LF (20 mg/ml), 4 ml of NaHCO_3_ (250 mM), and 1 ml of FeCl_3_·6H_2_O (10 mM) and incubating for 1 h followed by ultrafiltration desalination. The iron-saturated lactoferrin was dissolved in buffers of different pH (pH 8.0, 7.0, 6.0, 5.0, 4.0, 3.0, and 2.0) at 2 h after the detection of iron saturation for an indirect evaluation of the iron release capacity of LF. hLF and bLF were purchased from Sigma and Tianchun trade companies.

## Results

### Generation and identification of marker-free hLF BAC transgenic cows

**Figure 1:**
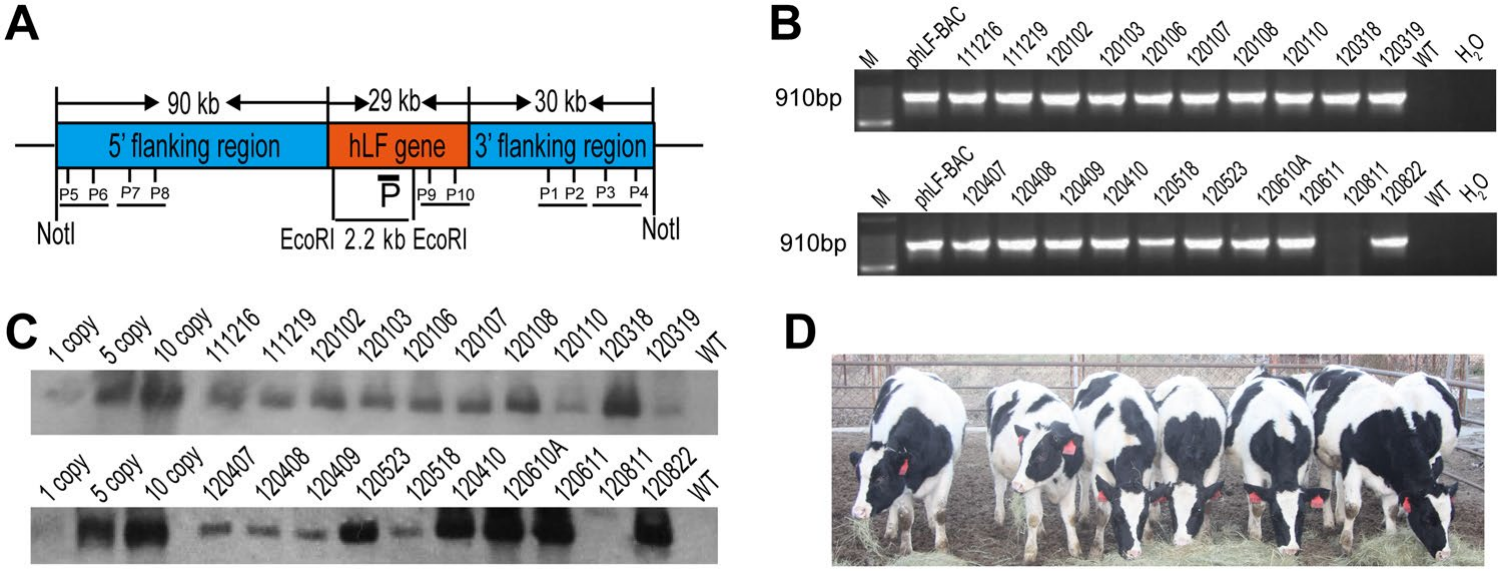
Generation of marker-free hLF BAC transgenic cloned cows. (**A**) Schematic representation of the transgene. The hLF BAC containing the complete hLF genomic DNA (a 29 kb genomic sequence consisting of human hLF flanked by a 90 kb 5′ flanking region and a 31 kb 3′ flanking region) was nucleofected into BFFs without a marker gene. The black box shows the 600 bp probe in Southern blotting upon digestion with EcoRI and a band of 2.2 kb resulting from the transgenic cloned cows. P1–P10 are five pairs of primers in different BAC regions for the identification of the transgenic cloned cows. (**B**) PCR analysis of transgenic cloned cows. Primers P1 and P2 amplified a 910 bp product to confirm positive transgenic cloned cows. M, 100 bp DNA ladder; pBAC-hLF, positive control; lanes 3–12, genomic DNA from transgenic cloned cows; H_2_O and WT, negative controls. (**C**) Southern blot identification of transgenic cloned cows. When digested with EcoRI, a band of 2.2 kb is detected in transgenic cloned cows. Lanes 1–3, positive controls with 1, 5 and 10 copies; Lanes 4–12, genomic DNA from transgenic cloned cows; WT, wild-type cow. (**D**) Image of transgenic cloned cows. The image shows transgenic cloned cows at the age of 10 months.

The low transfection efficiency of primary cells has always been a challenge in transgenic livestock research, especially for the transfection of large vectors such as BACs and yeast artificial chromosomes (YACs). Recently, it was reported that some primary cells could be efficiently transfected by nucleofection^19^. Our previous studies have demonstrated that the ZFN plasmid and mRNA can also be efficiently transfected into bovine primary cells by nucleofection^15^. Thus, in our study, nucleofection was used to transfect the BAC vector into bovine primary cells. To avoid the use of a selectable marker gene, only the 150 kb hLF gene fragment from the pBAC-hLF vector was nucleofected into three BFF cell lines, 0901FFB, 0904FFB and 1003FFB (Fig. 1A). After culturing by single-cell amplification, we isolated 375 0901FFB colonies, 577 0904FFB colonies and 315 1003FFB colonies. PCR analysis confirmed the presence of 16 positive colonies, of which six (#4, #17, #18, #20, #22, and #35) were used as donor cells for nuclear transfer (Table 1). As shown in Table 2, we produced 4,130 reconstructed embryos from these different colonies. Of these embryos, 47.5%, 30%, 39.2%, 38.1%, 46.3%, and 44.7% of those from colonies #4, #17, #18, #20, #22, and #35, respectively, successfully entered the blastocyst stage. In total, we transferred 492 transgenic cloned blastocysts into 328 recipients. A total of 30 cloned animals were born, but 10 of the 30 cloned animals died after birth due to the commonly observed effects of somatic cell cloning (dysfunction of lungs or kidneys). Ultimately, twenty cloned calves were live and normal. The transgenic cloned calves were confirmed by PCR analysis. Five pairs of primers were designed to detect the different regions of the BAC, including the 5′ region, hLF gene and 3′ region (Fig. 1A). As shown in Fig. 1B and Supplementary Fig. S1, nineteen of the twenty cloned cows were positive, while one cloned cow (#120811) was non-transgenic. These transgenic cows were further verified by Southern blot analysis (Fig. 1C). The signals of the bands from different samples revealed great variation in transgene copy numbers in the transgenic cloned cows. We also used qPCR to estimate the copy number, and the results indicated that the transgene copy numbers in the founders were different and ranged from 2 to 18. Images of some of the transgenic cloned cows are shown in Fig. 1D.

**Table 1:** Cell screening of pBAC-hLF transfection.

Cell line	Culture method	Isolated colonies	PCR positive cell colonies	Freezing cell colonies	Cell colonies for NT
0901FFB	Single cell culture	375	5	5	#18, #22, #35
0904FFB	Single cell culture	577	7	7	#4
1003FFB	Single cell culture	315	4	4	#17, #20

**Table 2:** Summary of nuclear transfer results.

Cell clone	Oocytes	Reconstruct embryo	Blastocysts	Blastocysts rate %	Recipients	Pregnancy day 60	Born	Live
0904FFB 4#	917	526	250	47.5	53	11	2	0
1003FFB 17#	1684	1074	322	30	38	9	3	2
1003FFB 20#	897	617	235	38.1	47	8	5	3
0901FFB 18#	1728	640	251	39.2	51	11	4	2
0901FFB 22#	1500	533	247	46.3	59	13	6	6
0901FFB 35#	1186	740	331	44.7	80	21	10	7

### Expression of rhLF in the milk of marker-free transgenic cows

**Figure 2:**
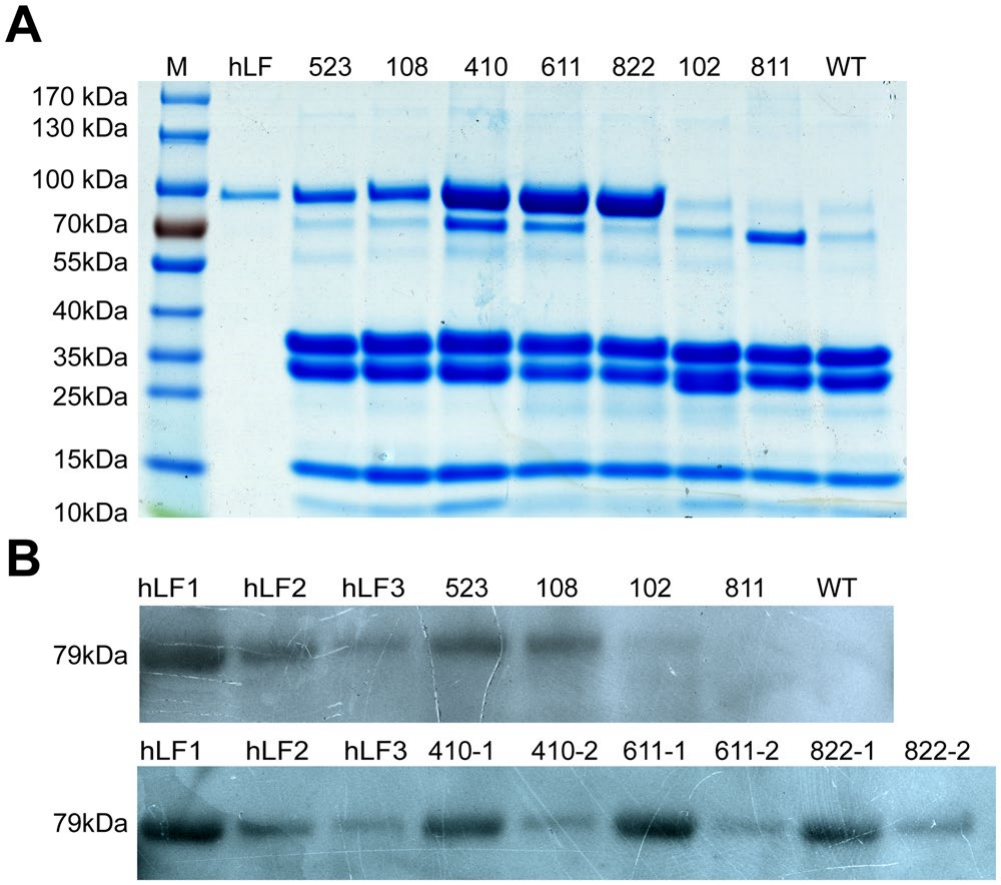
Expression of rhLF in milk. (**A**) SDS-PAGE analysis of milk from transgenic cloned cows. Milk samples from transgenic cloned cows were collected and detected by SDS-PAGE. hLF, 1 µg of commercial hLF (Sigma) as a positive control; 523, 108, 410, 611, 822, 102, and 811, 0.5 µl of raw milk from transgenic cloned cows; WT, 0.5 µl of raw milk from wild-type cows. (**B**) Identification of rhLF in milk by western blotting. hLF1, 10 µg of commercial hLF standard (mother solution concentration: 10 µg/µl, undiluted, 1 µl); hLF2, 5 µg of commercial hLF standard (mother solution concentration: 10 µg/µl, dilution 1:2, 1 µl); hLF3, 1 µg of commercial hLF standard (mother solution concentration: 10 µg/µl, dilution 1:10, 1 µl); 523, 108, 102, and 811, 1 µl of raw milk from transgenic cloned cows; 410-1, 611-1, 822-1, 1 µl of milk from transgenic cloned cows; 410-2, 611-2, 822-2, diluted milk from transgenic cloned cows (dilution, 1:10, 1 µl); WT, 1 µl of raw milk from wild-type cows.

To detect the expression of hLF in the milk of the transgenic cloned cows, SDS-PAGE and western blot analyses were performed. Milk samples from seven transgenic cloned cows and non-transgenic negative control cows were collected. An hLF standard obtained from Sigma was used as a positive control. The results of SDS-PAGE showed obvious rhLF bands in 120410, 120822 and 120611 but no bands in 120811 (Fig. 2A); this result was in accordance with the PCR and Southern blotting results. Western blot analysis showed positive bands for the samples from the transgenic cloned cows (Fig. 2B). An ELISA was used to quantify the rhLF expression levels in the milk of the transgenic cloned cows. As shown in Table 3, the rhLF concentrations in milk from 120822 and 120611 were the highest at 13.6 g/L and 11.2 g/L, respectively. These results suggested that expression levels correlated with BAC transgene copy number, a finding that was consistent with the results of a previous study^20, 21^. Additionally, to determine whether the transgenic cows had leaky expression of hLF in other organs, an RT-PCR assay was used to detect the transcription of hLF in various organs including the mammary gland, heart, liver, spleen, lung, kidney, large intestine and small intestine. As shown in Supplementary Fig. S2A, the expression of hLF was mammary-gland-specific. Subsequently, we also detected the expression of hLF at the protein level by SDS-PAGE and western blot analyses. As shown in Supplementary Fig. S2B and C, the hLF protein was found only in the mammary gland and not in other organs.

**Table 3:** Expression levels of recombinant hLF in milk from transgenic cloned cows.

The expression levels of rhLF					
No.	811	523	410	822	611
rhLF (g/L)	0	4.5	8.3	13.6	11.2

### Increased total protein in milk from marker-free transgenic cows

**Figure 3:**
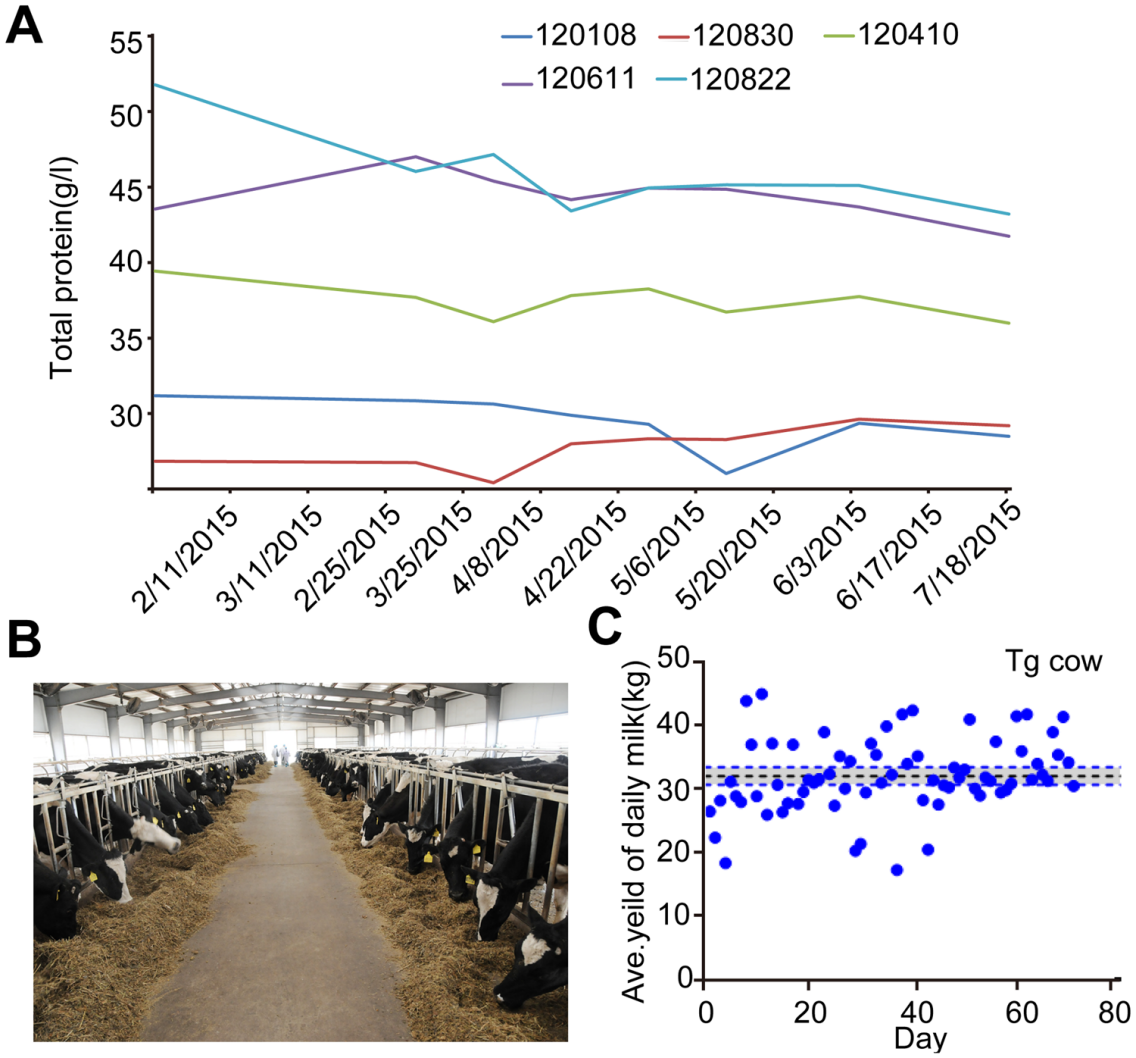
Detection of total protein in milk from transgenic cloned cows. (**A**) Identification of the total protein from different cows at different times. 120830 was a WT cow, and 120108, 120410, 120611 and 120822 were marker-free hLF BAC transgenic cloned cows. (**B**) Breeding of more than 200 transgenic cloned cattle. (**C**) Evaluation of the milk yield of the transgenic cloned cows.

Previous mouse research showed that the overexpression of BLG in transgenic mice corresponded with reduced endogenous protein levels, reflecting what appeared to be a ceiling to total milk protein production^12^. Similar results were also reported in both cows and sheep^9, 13^. In the present study, the total protein in the milk of the marker-free rhLF transgenic cows was determined using the MilkoScan^TM^ Minor analyser. We detected the total protein in milk from transgenic cows with five different rhLF expression levels at different lactation times using the WT 120830 cow as a control. As shown in Fig. 3A, the total milk protein was increased in the high-expressing 120822 animal. This result showed that a high level of rhLF expression did not significantly reduce the production of the endogenous protein, at least in some marker-free transgenic cloned cow lines. This result also suggested that the bovine mammary bioreactor may have a greater tolerance for protein expression. For larger-scale production of rhLF, more transgenic cows were needed. The MOET method^22^ was used to multiply the progeny of the 19 F_0_ female cows. As shown in Table 4, 910 embryos were produced by superovulation and artificial insemination from 2013 to 2016, and embryo transfer was subsequently performed to obtain 364 F_1_ cows, of which 178 were transgenic cows. The F_1_ offspring of four founders of lines 523, 410, 822 and 611 were analysed by PCR to determine BAC integrity. Four pairs of primers were designed to detect the different regions of the BAC, including the 5′ end, hLF gene and 3′ end (Fig. 1A and Supplementary Fig. S3). Additionally, we used qPCR to estimate transgene copy number in the F_1_ offspring. As shown in Supplementary Table S1, the copy numbers of F_1_ progeny were similar to those of their respective founders, which suggested that the transgenes may integrate into a single site as concatemers containing multiple copies of the BAC. The transgene copy numbers of some cows are shown in Fig. 3B. The milk yield of the transgenic cows was not significantly altered; the average milk yield was nearly 32 kg/d (Fig. 3C).

**Table 4:** Summary of MOET results.

F_0_ female (2011–2012)	Embryo production (2013–2016)	F_1_ cow (NT) (2013–2016)		In total (F_0_ + F_1_) Cow
		born	Transgenic cow	
19	910	364	178	197

### Large-scale purification of rhLF

**Figure 4:**
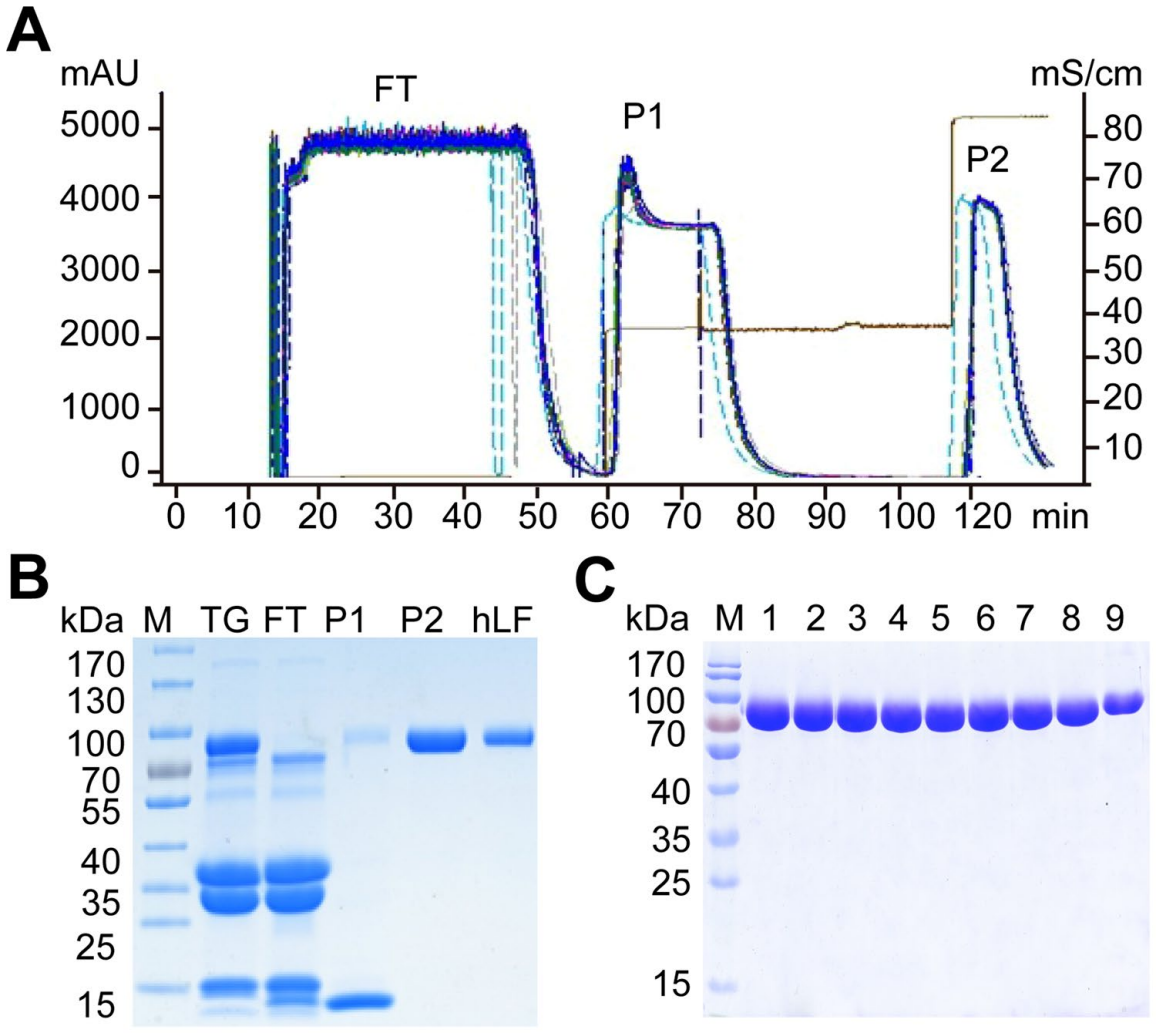
Identification of purified rhLF from transgenic milk. (**A**) Purification of rhLF by cation-exchange chromatography using a BPG 300/500 column. One hundred litres of milk from transgenic cloned cows was loaded onto a BPG 300/500 column. Peaks 1–3, proteins eluted from the column; only the protein from peak 2 was collected. (**B**) Identification of rhLF by SDS-PAGE. hLF was loaded as a standard. M, protein marker; hLF, human lactoferrin standard; TG, milk samples from transgenic cows; P1 and P2, rhLF eluted from the column; FT, protein fraction that was not bound to the column. (**C**) Identification of different batches of rhLF by SDS-PAGE. M, protein marker; 1–9, different batches of purified hLF.

The large-scale separation and purification of rhLF from transgenic cloned cow milk was based on the cation-exchange chromatography method^6^, which included three main steps: the removal of fat, the removal of casein and ion exchange. The fats were removed by centrifugation, and the casein proteins were removed by acid precipitation. The pretreated milk (100 L) was applied to a BPG 300/500 column to bind basic proteins, and the column was washed using 0.40 M and 1.0 M NaCl. Two proteins, P1 and P2, were eluted at different times by 0.40 M and 1 M NaCl, respectively (Fig. 4A). To determine the characteristics of the eluted proteins, protein peaks were detected by SDS-PAGE (Fig. 4B), and to further purify rhLF, the collected fraction was concentrated using an Ultracel-30 membrane. We purified nine batches of rhLF, which were identified by SDS-PAGE using the same procedure (Fig. 4C). Using this procedure, we obtained 400–450 g of rhLF in one day.

### Iron binding and release activity of rhLF

**Figure 5:**
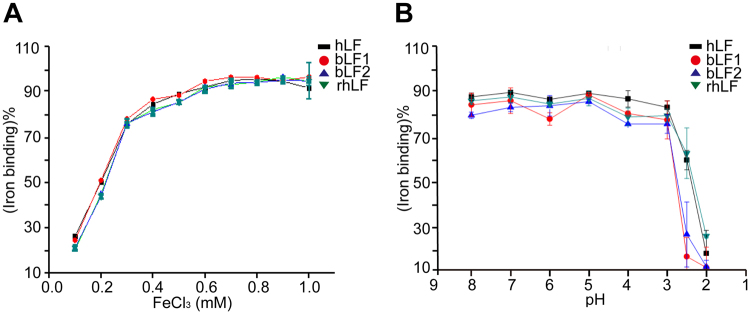
Iron binding and releasing properties of rhLF. (**A**) Profiles of iron binding by rhLF (green), Sigma-hLF (black), company1-bLF1 (red) and company1-bLF2 (blue) as a function of Fe concentration. (**B**) Profiles of iron release by rhLF (green), Sigma-hLF (black), Tianchun-bLF1 (red) and Tianchun-bLF2 (blue) as a function of pH.

Previous reports have shown that rhLF has similar iron binding and releasing capabilities to those of natural hLF^5, 6^. Therefore, iron binding and releasing experiments were also performed to determine the bioactivity of the rhLF purified from the milk of the marker-free transgenic cows. Different concentrations of iron were used to detect the iron-binding activity of the rhLF, with commercialized hLF, bLF1 and bLF2 as controls. As shown in Fig. 5A, our rhLF has similar iron-binding activity to the controls. We also analysed the iron-releasing properties of rhLF and compared them to native hLF, bLF1 and bLF2 at different pH values. The iron release from rhLF was also similar to those of the controls; release began at pH 4.5 and was complete at pH 2.0 (Fig. 5B).

## Discussion

In this study, we generated a herd of marker-free hLF BAC transgenic cloned cows that produced rhLF in their milk at levels from 4.5–13.6 g/L. To the best of our knowledge, no previous report has described rhLF concentrations in excess of 4.0 g in the milk of transgenic cows. The total protein in the milk was also increased. A total of 400–450 g of functional rhLF was successfully purified per day. Finally, we established a herd of two hundred rhLF BAC transgenic cloned cows by MOET, which provides a solid foundation for the large-scale production of rhLF in the future.

Thus far, the expression level of rhLF in our study is the highest reported. rhLF was first produced in transgenic bovine mammary glands in 1991, but in that study, the hLF gene cDNA was under the regulatory control of the bovine αS1-casein promoter. However, the expression of rhLF was not shown in that line^23^. In 2002, a bovine mammary bioreactor secreting functional rhLF at 2.8 g/L was established by the same group^5^. However, the limitations of using transgenic cattle as bioreactors have been the inefficiency of microinjection for generating founders and lengthy timelines. Recently, two groups have efficiently generated transgenic cattle by cytoplasmic injection using two transposon systems (Sleeping Beauty and piggyBac)^24, 25^. Importantly, multiply transgenic cattle were also generated by this technique, allowing for the generation of complex transgenic genotypes in the cattle model within a reasonable time frame^24^. These studies provide strong technological support for the use of transgenic cattle as mammary bioreactors.

Because BAC transgenes generally diminish the “position effect”, they promote high and sustained expression levels^20^. Thus, in our previous study^6^, we co-injected the 150 kb hLF BAC and a marker gene into donor cells by microinjection and combined that technique with SCNT to produce two transgenic cloned cows. The rhLF concentration in the resulting milk was 3.4 g/L, which was slightly higher than in previous studies^5^. This low expression of rhLF may be due to the introduction of antibiotic resistance genes into transgenic animals, which has many side effects, such as the abnormal regulation of adjacent genes^10^ and the genes of interest^11^. Importantly, a linear relationship has been reported between transgene BAC copy number and expression level^20^. However, the copy number of the hLF BAC was low in our previous study, which may have been due to the use of microinjection. Because some primary cells have been efficiently transfected via nucleofection^14^, our current study used a simple and efficient method to generate high-copy hLF BAC marker-free transgenic cloned cows by nucleofection. Thus, our transgenic cows contained many copies of the gene, resulting in the expression of a very high level of rhLF, 13.6 g/L. Notably, the introduction of antibiotic resistance genes into transgenic animals, as in previous reports, may arouse public concern about biological safety and complicate the approval of the produced recombinant proteins by the Food and Drug Administration (FDA) and European Medicine Agency (EMA)^26^. Our approach is more suitable for future applications of transgenic bovine mammary bioreactors. rhLF has been successfully expressed in fungi^27^, insect cells^28, 29^ and mammal cells^30^. However, some disadvantages in these systems, such as inaccurate posttranslational modification, slow production, low expression and high cost, affect the applications of the resulting rhLF. Thus, these systems present inherent difficulties for large-scale production. Recent advances in cell culture technology for CHO cells have achieved significant improvements in the production of some proteins, leading to titres of more than 10 g/L to meet the huge market demand^31^. However, in contrast to the transgenic bovine mammary bioreactor, the cost of the CHO system is high and requires more professional and technical personnel for operation. For these reasons, the transgenic bovine mammary bioreactor is considered ideal for large-scale production of heterologous proteins due to its high protein synthesis capacity and efficient secretion and the low feed and housing costs of dairy animals.

Although the transgenic cloned cows consistently expressed extremely high levels of rhLF in their milk, there were no apparent adverse effects on the cows; the physiological and biochemical indicators were all within the normal range, and the cows remained alive. We also found that the transgenic cloned cows did not show downregulation of endogenous milk proteins; rather, the total protein was significantly increased. Additionally, the milk yield was not apparently altered. These results are in contrast to the findings of other studies^9, 12, 13^ and suggest that the bovine bioreactor may have greater potential for application in the large-scale production of functional recombinant proteins. This recombinant protein can be harvested on a large scale (>100 L milk) each day. Thus, enormous quantities of this biological protein could be produced by our group every year.

Many recent reports have described the effects of the consumption of milk containing rhLF and rhLZ (recombination human lysozyme) on both human health (based on animal models) and as treatments in animal models of disease^17, 32–34^. Importantly, transgenic cows with a high concentration of hLF in their milk seemed to be protected from clinical diseases and from prolonged inflammatory reactions. The high levels of rhLF (4.5–13.6 g/L) produced in the milk of our marker-free transgenic cloned cows may actually improve the animals’ health. Additionally, the development of SCNT and genome-editing technology^15, 16, 35–37^ may accelerate the application of transgenic bovine mammary glands.

In summary, we have established a convenient method to produce marker-free hLF BAC transgenic cloned cows that express high levels of functional rhLF. We also established two hundred transgenic cloned cows for the large-scale production of rhLF in the future. Taken together, these data lay the foundation for an important system for the large-scale production of biopharmaceutical proteins.

## Electronic supplementary material

Supplementary information
