# Supplementary material for: Large-scale production of recombinant human lactoferrin from high-expression, marker-free transgenic cloned cows
Source: Sci Rep. 2017 Sep 6;7:10733. doi: 10.1038/s41598-017-11462-z (PMC5587717; doi:10.1038/s41598-017-11462-z)
Supplement: Supplementary file 1 — Supplementary information [file 41598_2017_11462_MOESM1_ESM.pdf]

SUPPLEMENTARY INFORMATION

Large-scale production of recombinant human lactoferrin from high-expression, marker-free transgenic cloned cows

Ming Wang[1,4], Zhaolin Sun[1,4], Tian Yu[2,4], Fangrong Ding[1], Ling Li[1], Xi Wang[2], Mingbo Fu[2], Haiping Wang[1], Jinming Huang[3], Ning Li[1*], Yunping Dai[1*]

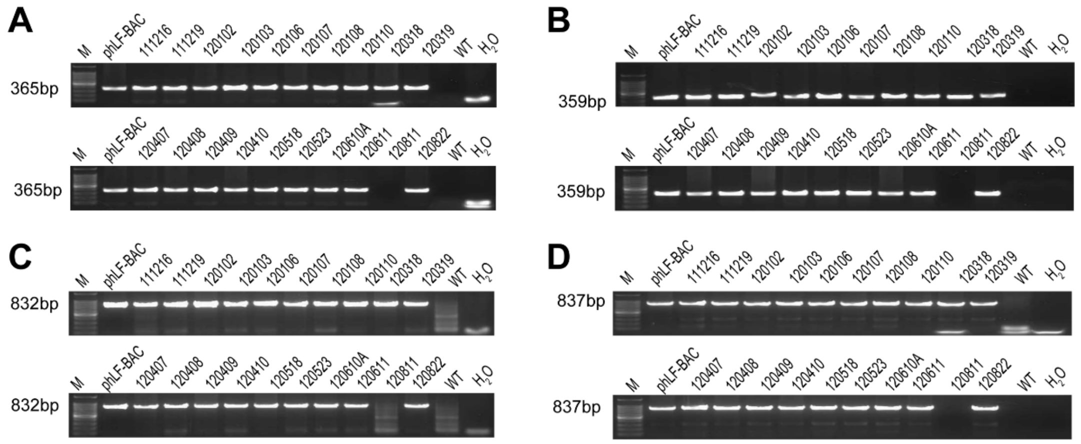

Fig S1. Identification of the hLF BAC transgenic cloned cows. (A) PCR analysis of transgenic cloned cows. P3-P4 primers was used to amplify a 365 bp product. M, 100 bp DNA ladder; pBAC-hLF, positive control; lanes 3–12, genomic DNA from transgenic cloned cows; H_2O and WT, negative controls. (B) PCR analysis of transgenic cloned cows. P5-P6 primers was used to amplify a 359 bp product. M, 100 bp DNA ladder; pBAC-hLF, positive control; lanes 3–12, genomic DNA from transgenic cloned cows; H_2O and WT, negative controls. (C) PCR analysis of transgenic cloned cows. P7-P8 primers was used to amplify an 832 bp product. M, 100 bp DNA ladder; pBAC-hLF, positive control; lanes 3–12, genomic DNA from transgenic cloned cows; H_2O and WT, negative controls. (D) PCR analysis of transgenic cloned cows. P9-P10 primers was used to amplify an 837 bp product. M, 100 bp DNA ladder; pBAC-hLF, positive control; lanes 3–12, genomic DNA from transgenic cloned cows; H_2O and WT,

negative controls.

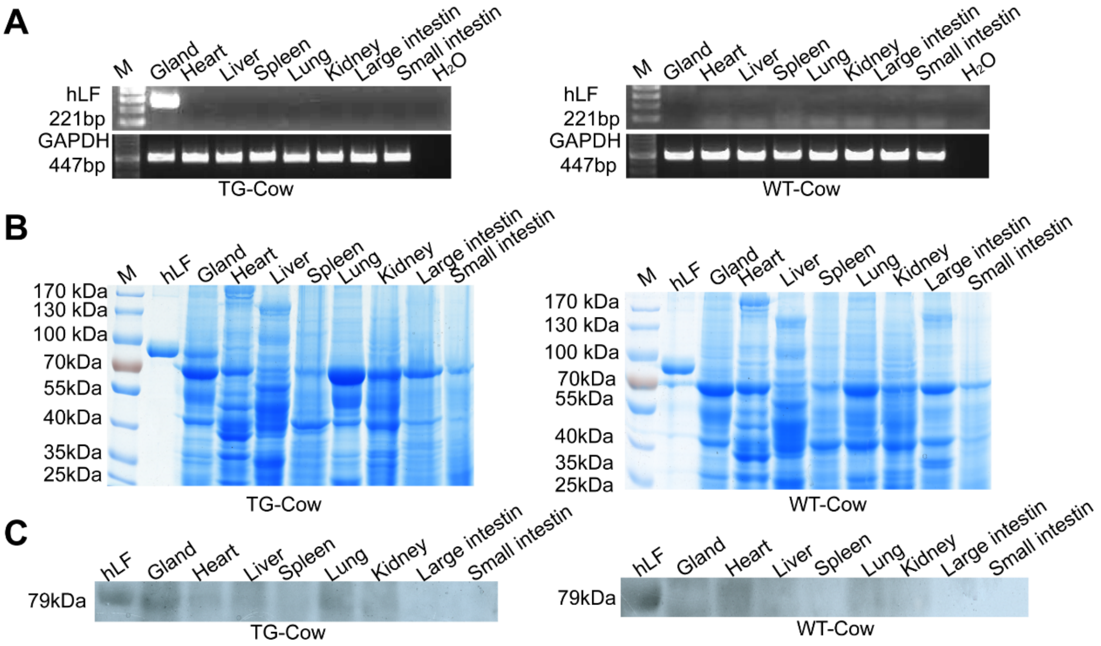

Fig S2. Identification of the hLF gland-specific expression. (A) Detection of the expression of the hLF in various organs by RT-PCR. For RT-PCR, the designed primers (RT-UP:5' TAGGAGGAG TGTTCAGTGGT 3'; RT-DOWN: AGGTCGCAGTTTGTAGGG) annealed in exon 2 and exon 3 and amplified a correctly spliced product of 221 bp. WT-cow, the samples of the wild-type; TG-cow, the samples of the hLF transgenic cow; M, 100 bp DNA ladder; lanes 2–10, cDNA from various organs of the transgenic cloned cow; H_2O, negative controls. Bovine GAPDH was used as a control (477 bp) (G1: 5' GCAAGTTCCACGGCACAG 3'; G2: CGCCAGTAGAAGCAGGGAT). (B) SDS-PAGE analysis of hLF protein expression in various organs. WT-cow, the samples of the wild-type; TG-cow, the sample of the hLF transgenic cow; M, (10 – 170 kDa) protein ladder; hLF, 5 µg of commercial hLF (Sigma) as the positive control; lanes3–10, 80 µg proteins from various organs of the transgenic cloned cow. (C) Western-blot analysis of hLF protein expression in various organs. WT-cow, the samples of the wild-type; TG-cow, the samples of the hLF transgenic cow; M, (10 -170 kDa)

protein ladder; hLF, 5 μg of commercial hLF (Sigma) as the positive control; lanes 2–9, 80 μg proteins from various organs of the transgenic cloned cow;

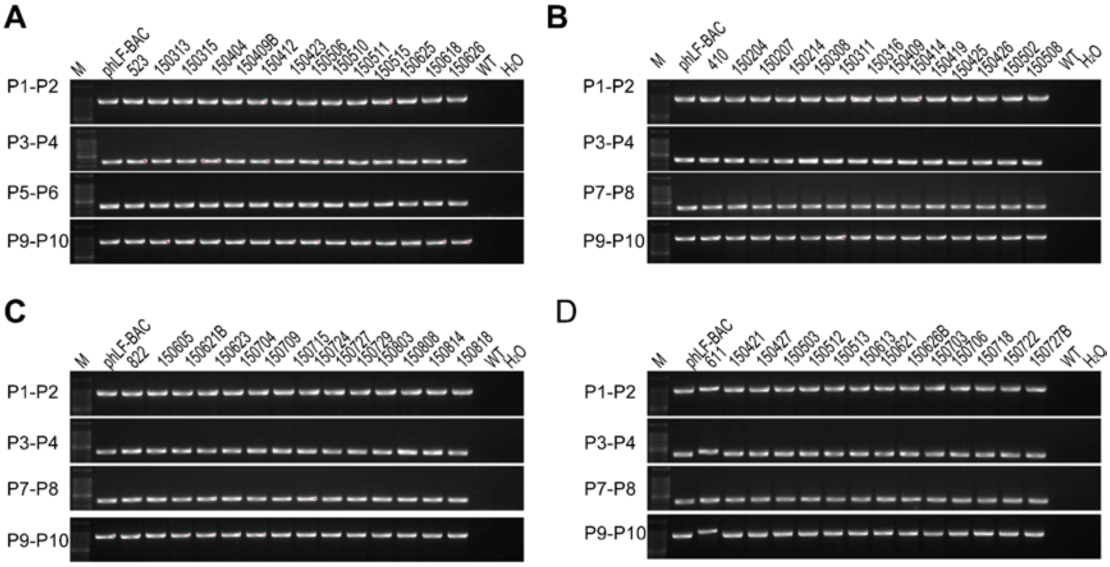

Fig S3. Identification of the F_1 transgenic cloned cows. (A) PCR for detecting F_1 transgenic cloned cows from 523 TG founders. Four pairs of primers were used to amplify 910 bp, 365 bp, 359 bp and 837 bp products, respectively. M, 100 bp DNA ladder; pBAC-hLF, positive control; lanes 3, 523 TG founders; lanes 4–12, genomic DNA from F_1 transgenic cloned cows; H_2O and WT, negative controls. (B) PCR for detecting F_1 transgenic cloned cows from 410 TG founders. Four pairs of primers were used to amplify 910 bp, 365 bp, 359 bp and 837 bp products, respectively. M, 100 bp DNA ladder; pBAC-hLF, positive control; lanes 3, 410 TG founders; lanes 4–12, genomic DNA from F_1 transgenic cloned cows; H_2O and WT, negative controls. (C) PCR for detecting F_1 transgenic cloned cows from 822 TG founders. Four pairs of primers were used to amplify 910 bp, 365 bp, 359 bp and 837 bp products, respectively. M, 100 bp DNA ladder; pBAC-hLF, positive control; lanes 3, 822 TG founders; lanes 4–12, genomic DNA from F_1 transgenic cloned cows; H_2O and WT, negative controls. (D) PCR for detecting F_1 transgenic cloned cows from 611 TG founders. Four pairs of primers were used to

amplify 910 bp, 365 bp, 359 bp and 837 bp products, respectively. M, 100 bp DNA ladder; pBAC-hLF, positive control; lanes 3, 611 TG founders; lanes 4–12, genomic DNA from F_1 transgenic cloned cows; H_2O and WT, negative controls.

Table S1. The copy number of the F_1 transgenic cows.

F_0	Copy number	F_1	Copy number
120523	13.02483	150313	13.28799
		150315	13.31173
		150404	13.69493
		150409B	13.45487
		150412	13.57463
		150423	12.9105
		150506	13.85433
		150510	13.04285
		150511	13.85433
		150515	13.38764
		150625	13.57333
		150618	13.2031
		150626	13.64331
120410	16.7135	150204	16.36924
		150207	17.38555
		150214	16.44836
		150308	17.22297
		150311	16.44836
		150316	16.79671
		150409	16.69598
		150414	16.74624
		150419	17.20243
		150425	17.15143
		150426	17.48414
		150502	17.02435
		150508	18.13307
120822	19.59851	150605	18.30441
		150621B	18.69551
		150623	18.30419
		150704	18.01977
		150709	17.25539
		150715	17.38165
		150724	18.20771
		150727	19.68091
		150729	19.05792

		150803	18.44486
		150808	19.9549
		150814	18.2603
		150818	21.18311
120611	17.55218	150421	18.22614
		150427	17.56611
		150503	17.27952
		150512	18.22614
		150513	16.73898
		150613	16.99528
		150621	18.12605
		150626B	17.00992
		150703	17.33293
		150706	17.90552
		150718	17.82316
		150722	17.96057
		150727B	17.09039
